# Geographic Pattern of Variations in Chemical Composition and Nutritional Value of *Cinnamomum camphora* Seed Kernels from China

**DOI:** 10.3390/foods12132630

**Published:** 2023-07-07

**Authors:** Xianghui Yan, Xiaofeng Gong, Zheling Zeng, Jiaheng Xia, Maomao Ma, Junxin Zhao, Guohua Zhang, Pengbo Wang, Dongman Wan, Ping Yu, Deming Gong

**Affiliations:** 1 State Key Laboratory of Food Science and Resources, Nanchang University, Nanchang 330047, China; xianghui_y@163.com (X.Y.); xiajiaheng@ncu.edu.cn (J.X.); ma_maomao@hotmail.com (M.M.); junxinzhao@hotmail.com (J.Z.); zhangguohua2050@163.com (G.Z.); 5804117050@email.ncu.edu.cn (P.W.); yuping@ncu.edu.cn (P.Y.); 2 Jiangxi Province Key Laboratory of Edible and Medicinal Resources Exploitation, Nanchang University, Nanchang 330031, China; 3 School of Resources and Environment, Nanchang University, Nanchang 330031, China; xfgong@ncu.edu.cn; 4 School of Chemistry and Chemical Engineering, Nanchang University, Nanchang 330031, China; 5 School of Food Science and Technology, Nanchang University, Nanchang 330031, China; dmwanjx@ncu.edu.cn; 6 Institute of Biological Resources, Jiangxi Academy of Sciences, Nanchang 330096, China; 7 New Zealand Institute of Natural Medicine Research, 8 Ha Crescent, Auckland 2104, New Zealand

**Keywords:** *Cinnamomum camphora* seed kernel, camphor tree, medium-chain oil, mineral, essential amino acid

## Abstract

*Cinnamomum camphora* (camphor tree) is an important non-conventional edible plant species found in East Asia. Here, a detailed characterization for the chemical composition and nutritional value of *C. camphora* seed kernels (CCSKs) collected from different regions in China is provided. The results showed that there were significant differences among the CCSK samples in weights (1000 fruits, 1000 seeds and 1000 kernels), proximate composition, minerals, phenolics, flavonoids and amino acid contents. The highest contents of oil (62.08%) and protein (22.17%) were found in the CCSK samples collected from Chongqing and Shanghai, respectively. The highest content of mineral in the CCSK samples was K (4345.05–7186.89 mg/kg), followed by P (2735.86–5385.36 mg/kg), Ca (1412.27–3327.37 mg/kg) and Mg (2028.65–3147.32 mg/kg). The CCSK sample collected from Guizhou had the highest levels of total phenolic and flavonoid contents (TPC and TFC), while that from Chongqing had the lowest levels. In addition, the most abundant fatty acid in the CCSK samples was capric acid (57.37–60.18%), followed by lauric acid (35.23–38.29%). Similarities in the fatty acid composition among the CCSK samples were found. The CCSK sample collected from Guizhou had the highest percentage (36.20%) of essential amino acids to total amino acids, and Chongqing had the lowest value (28.84%). These results indicated that CCSK may be developed as an excellent source of plant-based medium-chain oil, protein, dietary fiber, minerals, phytochemicals and essential amino acids.

## 1. Introduction

Of the thousands of edible plant species known on Earth, only 150 to 200 species are utilized by humans, both for direct consumption and to produce food ingredients such as oils, proteins, spices, etc. [1]. Many plants with food potential have yet to be developed and are known as “non-conventional edible plants” (NCEPs). Recently, these NCEPs are becoming increasingly important, especially considering that the demand for food supplies will increase dramatically due to population growth. *Cinnamomum camphora* (L.) Presl, the plant of *Lauraceae*, commonly known as camphor tree, is widely distributed in East Asia [2]. In particular, as a massive broad-leaved evergreen tree with a broad sweeping crown, *C. camphora* is widely cultivated as a garden tree and street tree in the southern region of the Yangtze River in China [3]. *C. camphora* has long been known for its great potential applications in the food industry for the essential oils extracted from its roots, barks, branches, leaves and fruits [4,5,6,7]. Additionally, it was reported that the annual yield of *C. camphora* seeds in China was over one million tons [3]. Specifically, the hot-pressed oil from *C. camphora* seeds has been used as a cooking oil in China’s Anfu County since the 1960s [8]. Therefore, it is of great significance to explore *C. camphora* seeds as sustainable and efficient alternative food sources.

*C. camphora* seeds comprise an outer shell and a kernel. In recent years, there has been a growing industry interest in *C. camphora* seed kernels (CCSKs) due to their excellent pharmacological and nutritional properties. The high nutritional value of CCSKs is attributed to its high content of oil (59.34%), proteins (19.34%), dietary fibers (10.91%) and phenolics (0.97%) [3]. In particular, CCSK oil is rich in fatty acids with 8–12 carbon chains (more than 95%), significantly higher than those of coconut oil (62%) and palm kernel oil (55%). Also, CCSK oil has been found to be safe and regulate glucose and lipid metabolism [8,9,10]. In this respect, the oil has the potential for use in structured lipids and pharmaceuticals. Moreover, the protein isolated from CCSK has been reported to have comparable nutritional value to soybean protein as it contains sufficient essential amino acids (EAA) [3]. The EAA in CCSK protein isolate meet the recommendations of the FAO and WHO for adults. Thus, CCSKs may be an excellent alternative source of plant protein. CCSKs are also a valuable source of phytochemical constituents, especially phenolics and flavonoids. An 80% ethanol extract from CCSKs exhibited various potential bioactive properties, including antioxidant, anti-diabetic, anti-obesity, anti-cholinergic and anti-inflammatory activities [2,11].

It should be mentioned that the chemical profiles of plants depend on many factors, including degree of maturity, growing location and weather conditions. For example, Liu et al. [12] reported that the yield of peony seeds and the content of the main fatty acids of peony seed oil significantly depended on the cultivation areas. Similarly, Chinese black truffle collected from different geographical regions showed varying degrees of nutritional value and biological activity [13]. *C. camphora* is mainly cultivated in China, including fourteen provinces (Sichuan, Yunnan, Guizhou, Chongqing, Guangxi, Hunan, Guangdong, Hubei, Anhui, Jiangxi, Fujian, Jiangsu, Zhejiang, Shanghai). Although there are previous studies describing the chemical compositions and bioactivities of CCSK, there is limited information on the samples collected from different geographical regions.

The study aimed to systematically investigate the physical characteristic (weights), chemical composition (oil, proteins, dietary fibers and ash) and nutritional value (minerals, phenolics, flavonoids, fatty acids and amino acids) of CCSK samples collected from different geographical regions in China. As a growing trend in the recycling of nutritional food ingredients from plants, this study will improve the chemical profiles and nutritional value of CCSKs and promote their effective utilization.

## 2. Materials and Methods

### 2.1. Collection of Plant Material

In China, the maturity of *C. camphora* fruits is the highest in December, and the amount of fallen fruits is also the largest. Hence, fourteen samples of *C. camphora* fruit were collected from the Yangtze River basin and its southern region in China from 1–31 December 2021. The geographical locations of the studied accessions are shown in Table 1. After being taken to the laboratory, the *C. camphora* fruits were manually cleaned, then all flesh was removed and dried at 35 °C for 48 h. Then, the seed shell was removed to obtain the CCSK and stored at 4 °C.

### 2.2. Chemical Reagents

Folin–Ciocalteu was purchased from Sigma-Aldrich Trading Co., Ltd. (Shanghai, China). Thirty-seven fatty acid methyl ester mixed standards were purchased from Shanghai Anpel Experimental Technology Co., Ltd. (Shanghai, China). Isopropyl alcohol, acetonitrile and methanol (HPLC-grade) were purchased from Thermo Fisher Technology Co. (Shanghai, China). All other solvents and reagents used were of analytical grade.

### 2.3. Measurement of Fruit, Seed and Kernel Weights

The weight of 1000 fruits (wet, W1) of the sample was immediately measured after being taken to the laboratory. The weight of 1000 seeds (wet, W2) was measured after removal of flesh and then the weight of 1000 seeds (dry, W3) was measured after dried. After the dry seed shell was removed, the weight of 1000 kernels (dry, W4) was obtained. Based on the above, the seed rate of the fruit (wet, R1) and kernel rate of the seed (dry, R2) were calculated [14].

R1 (%) = W2/W1 × 100%.(1)

R2 (%) = W4/W3 × 100%.(2)

### 2.4. Determination of Proximate Composition of CCSK

The proximate composition of CCSK, including oil, protein, dietary fiber, moisture and ash, was determined by using a series of Chinese National Standards. Lipid content was measured by using Soxhlet extraction (GB 5009.6—2016). Protein content was determined using the Kjeldahl method (N × 6.25) (GB 5009.5—2016). Dietary fiber content was determined using enzymatic hydrolysis, followed by the weighting method (GB 5009.88—2016). Moisture content was determined by using an HX204 Moisture Analyzer (METTLER TOLEDO, Zurich, Switzerland) according to GB 5009.3—2016. Ash content was evaluated via incineration of the sample at 550 °C for 4 h (GB 5009.4—2016).

### 2.5. Determination of Minerals and Heavy Metals of CCSK

The minerals and heavy metals in CCSKs were determined according to Chinese National Standard GB 5009.268—2016 by using an inductively coupled plasma optical emission spectrometer (ICP-OES 725ES, Agilent, Santa Clara, CA, USA). Prior to analysis, the samples were decomposed with 8 mL of nitric acid in a Microwave Digestion System (MARS6, CEM, Charlotte, NC, USA) for 1 h. Then, the digestion solution was transferred into a 100 mL volumetric flask, diluted with ddH_2_O to the volume. The main parameters of ICP-OES were set as follows: radio-frequency power, 1.20 KW; plasma gas flow, 15.0 L/min; auxiliary gas flow, 1.5 L/min; observed height, 10 mm; atomizer pressure, 240 KPa.

### 2.6. Determination of Phytochemical Constituents of CCSKs

#### 2.6.1. Preparation of Phytochemical Extracts

The extracts were prepared by mixing the defatted CCSKs with 80% aqueous–ethanol solution at a ratio of 1:20 (*w*/*v*) [3]. The mixture was stirred at room temperature for 2 h with the speed of 300 rpm and then filtered with an aspirator filter pump. The residue was re-extracted 5 times by using the above procedure. Both extracts were combined and stored at 4 °C for further analysis.

#### 2.6.2. Total Phenolic Content (TPC)

Folin–Ciocalteu method was used to determine the TPC [2]. Briefly, a 40 μL aliquot of the extract was mixed with 20 μL of Folin–Ciocalteu reagent and reacted at room temperature for 6 min. Then, 200 μL of 7% Na_2_CO_3_ was added to neutralize the reaction. After incubation at room temperature in the dark for 1 h, the absorbance value was measured at 760 nm using a microplate reader (ReadMax 1200, Flicker Biotechnology Co., Ltd., Shanghai, China). Results were expressed as gram of gallic acid equivalent (GAE) per 100 g of CCSK (g GAE/100 g).

#### 2.6.3. Total Flavonoid Content (TFC)

TFC was determined using the method of Tian et al. [15]. Briefly, a 2.5 mL aliquot of the extract was mixed with 300 μL of NaNO_2_ (5%) and incubated at room temperature for 6 min. Then, 300 μL of AlCl_3_ (5%) was added and incubated at room temperature for 6 min, followed by mixing with 4.4 mL of NaOH (4%), and incubation for 10 min. The absorbance value was measured at 510 nm. Results were expressed as gram of rutin equivalent (RE) per 100 g of CCSK (g RE/100 g).

#### 2.6.4. HPLC Profile

The Agilent 1260 HPLC system combined with a UV detector was used to analyze 80% ethanol extracts of CCSKs [16]. Prior to analysis, the extract solutions were filtered through 0.45 mm membrane filters. Samples (10 μL) were injected onto an amethyst (Sepax Technology Co., Ltd., Newark, DE, USA) C18-H reverse-phase column (250 mm × 4.6 mm × 5 μm) at 30 °C. The mobile phases consisted of 0.1% (*v*/*v*) formic acid in water (A) and acetonitrile (B). The gradient conditions were as follows: 15–40% B at 0–20 min; 40–50% B at 20–25 min; 50–95% B at 25–28 min; 95–15% B at 28–30 min. The flow rate was 1.0 mL/min and the absorbance was recorded at 280 nm.

### 2.7. Determination of In Vitro Antioxidant Activity

The DPPH free-radical-scavenging method was used to determine antioxidant activity [17]. The extract solution (150 μL, 1 mg/mL) was mixed with DPPH solution (150 μL, 0.1 mmol/L in 95% ethanol) and incubated in dark at room temperature for 30 min. The absorbance was measured at 517 nm by using a microplate reader (ReadMax 1200, Flicker Biotechnology Co., Ltd., Shanghai, China). The results were expressed as μmol of Trolox equivalent (TE) per gram of extract (μmol TE/g extract).

### 2.8. Determination of Fatty Acid Composition in Kernel Oil of CCSKs

Methyl esterification of the kernel oil was analyzed using an Agilent 7890B gas chromatograph with a DB-23 column (60 m × 250 μm × 0.25 μm, Agilent, Santa Clara, CA, USA) [18]. The major parameters were set as follows: carrier gas, nitrogen; flow rate, 2 mL/min; injection temperature, 250 °C; detection temperature, 250 °C. The column temperature was first kept at 50 °C for 1 min, increased to 175 °C at 20 °C/min and kept at 175 °C for 5 min, increased from to 230 °C at 4 °C/min and then kept at 230 °C for 10 min. The fatty acid profiles of the kernel oil were obtained by comparing the gas chromatograms of samples with thirty-seven fatty acid methyl ester mixed standards (Anpel, Shanghai, China) using normalization method.

### 2.9. Determination of Amino Acid Composition in Kernel Protein of CCSKs

The amino acid composition of CCSKs was determined using an amino acid auto-analyzer (S-433D, Sykam GmbH, Eresing, Germany) [19]. Prior to analysis, 0.1 g aliquot of each sample was mixed with 10 mL of HCl (6.0 M) and 1.0 g phenol in sealed glass tubes at 110 °C for 24 h. The hydrolyzed samples were filtered, placed in 50 mL volumetric flasks and diluted with ddH_2_O to the volume. The hydrolyzed sample (1 mL) was transferred to a glass dish and evaporated at 60 °C. Then, 4 mL of sample dilution buffer (0.12 N, pH 2.2) was added and filtered through a 0.22 μm PTFE filter.

### 2.10. Protein Profiles

The kernel protein extraction was carried out based on a previous study [3]. Sodium dodecyl sulphate–polyacrylamide gel electrophoresis (SDS-PAGE) under reducing condition was performed.

### 2.11. Statistical Analysis

All experiments were conducted in triplicate. Data were expressed as means ± standard deviation (SD) and subjected to one-way analysis of variance (ANOVA), followed by Tukey’s test for comparison of means (*p* < 0.05) using SPSS software. Principal component analysis (PCA) was performed in Origin 2023 software.

## 3. Results and Discussion

### 3.1. Physical Characteristics

#### 3.1.1. Shape and Color

**Figure 1:**
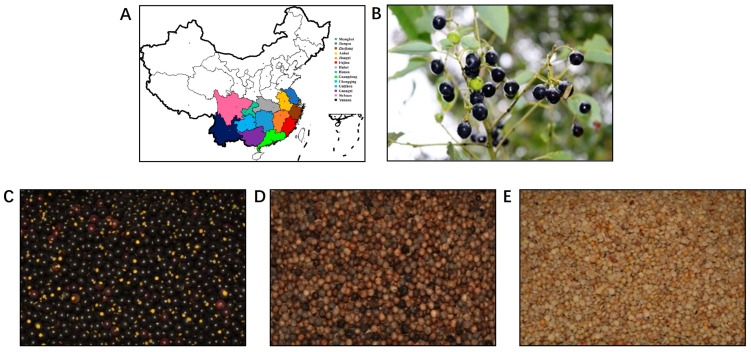
The distribution (**A**), plant (**B**), fruit (**C**), seed (**D**) and kernel (**E**) of *C. camphora*. A: different colors represent the *C. camphora* samples collected from different provinces of China, with each dot representing the provincial capital city of each province.

**Figure 2:**
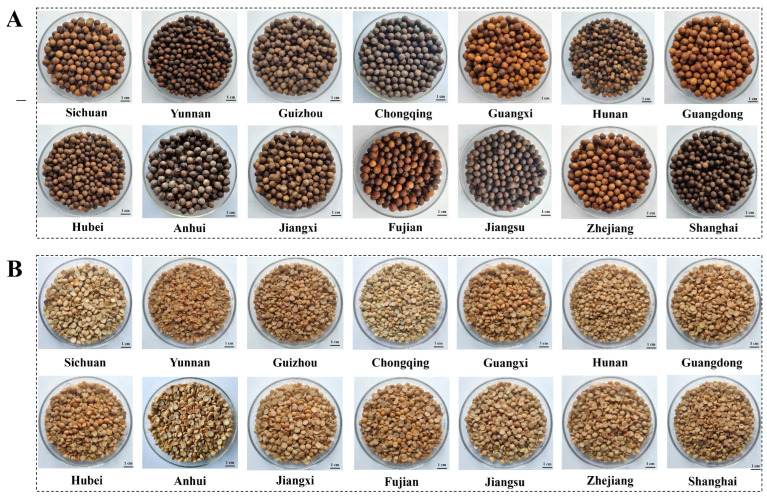
Comparative appearance of *C. camphora* seeds (**A**) and kernels (**B**) collected from different geographical regions.

In this study, *C. camphora* fruits were collected from different geographical regions in China (Table 1), followed by removal of the pulp and dehulling (Figure 1). The ripe *C. camphora* fruits were round, purplish-black in color and 7.30–11.26 mm in diameter. After removal of the pulp and dehulling, the CCSK was hemispherical, similar to yellow peas (Figure 1). All the *C. camphora* seeds from different geographical regions were round and smooth-faced (Figure 2A). Significant differences were observed in the shell colors between samples. There were three groups that can be used to classify the shell color of *C. camphora* seeds: yellow or yellowish (Zhejiang, Fujian, Guangdong, Guangxi, Sichuan), light brown (Jiangxi, Hubei, Hunan, Guizhou) and brown or darker (Shanghai, Jiangsu, Chongqing, Anhui, Yunnan), indicating the diversity of shell color of *C. camphora* seeds in nature. Nonetheless, the shell was removed during food processing in order to reduce or eliminate the anti-nutrients that affect the quality of the kernel oil and protein [20]. The colors of the CCSKs collected from different geographical regions were basically the same, ranging from light yellow to dark yellow (Figure 2B).

#### 3.1.2. Weights

As shown in Table 2, there were significant differences (*p* < 0.05) in the values of six weight indicators between samples. For the wet samples, *C. camphora* fruits collected from Guangdong had the highest value of W1 (896.87 g), followed by those from Guangxi (761.90 g) and Fujian (692.07 g), while Hunan and Yunan had the lowest values (339.03 g and 387.80 g, respectively). Guangxi had the highest value of W2 (262.90 g), followed by Guangdong (250.17 g) and Sichuan (241.47 g), and Hunan and Yunan had the lowest values (122.53 g and 114.57 g, respectively). In R1, Anhui was the highest (39.15%), followed by Jiangsu (37.36%), Sichuan (37.10%) and Zhejiang (36.62%), and Guangdong was the lowest (27.89%). For the dry samples, Guangxi had the highest W3 (189.60 g), followed by Guangdong (171.73 g) and Fujian (164.13 g), and Hunan and Yunan had the lowest values (81.77 g and 74.35 g, respectively). Sichuan had the highest W4 (114.04 g), followed by Jiangxi (100.99 g) and Zhejiang (100.05 g), and Yunan had the lowest value (42.97 g). In R2, Guizhou was the highest (79.76%), followed by Jiangxi (76.57%) and Hunan (75.19%), and Guangdong was the lowest (47.57%). Interestingly, Guangdong had the highest W1 (896.87 g) but the lowest R1 and R2 (27.89% and 47.57%, respectively). Although Hunan had the lower W1 to W4, its R1 and R2 were relatively high. It could also be observed that the pattern of change in W1 was basically consistent with W2 and W3, suggesting that the mass ratio of pulp (1-R1) and moisture content in different samples changed slightly. On the other hand, there were some differences between the patterns of change in W3 and W4, suggesting that the mass ratio of the shell (1-R2) varied greatly between different samples. The average values of the six weight indicators were 569.37 g, 195.48 g, 135.40 g, 84.60 g, 34.46% and 63.38%, respectively, meaning that the wet seed accounted for about 1/3 of the weight of the wet fruit, while the dry kernel accounted for about 2/3 of the weight of the dry seed.

### 3.2. Proximate Composition

CCSK samples were found to have high oil contents, with a mean value of 54.59 g/100 g, among which Chongqing was the highest (62.08 g/100 g), followed by Anhui (61.79 g/100 g) and Guizhou (57.89 g/100 g), and Hunan was the lowest (46.29 g/100 g) (Table 3). The oil contents in CCSKs were consistent with the previous reports [3,21]. As a natural medium-chain triglyceride (MCFA > 95%), the oil showed great potential in preventing obesity, inflammation, cardiovascular disease and diabetes [9,10,22]. In addition, CCSKs collected from Shanghai had the highest protein content (22.17 g/100 g), followed by those from Guangdong (20.07 g/100 g) and Jiangsu (19.83 g/100 g), and those from Yunan had the lowest content (12.33 g/100 g). The contents of moisture, dietary fiber and ash were basically the same among samples (1.62–3.28 g/100 g, 5.66–8.03 g/100 g and 2.15–2.96 g/100 g, respectively). CCSKs rich in dietary fiber have a positive effect on health [23]. The determination of moisture content is essential for assessing food quality, preservation and resistance to spoilage [24]. Overall, the results revealed that CCSKs could be used as potential sources of plant-based oil, protein and dietary fiber.

### 3.3. Minerals and Heavy Metals

Mineral elements play an important role in normal functioning of the human body [25]. To the best of our knowledge, the mineral and heavy metal contents in CCSKs have not yet been evaluated. As shown in Table 4, all the CCSK samples had high mineral contents, with a great variation among them. Among them, Ca, K, Mg and P were the main mineral elements in CCSK samples, which varied from 1412.27 to 4573.18 mg/kg, 4345.05 to 7186.89 mg/kg, 2158.52 to 3147.32 mg/kg and 2735.86 to 5385.36 mg/kg, respectively. The high contents of Ca, K, Mg and P are important quality feature of CCSKs, since Ca, Mg and P are essential part of bones and teeth; K and Mg also play important roles in maintaining cellular metabolism [26]. CCSKs collected from Hunan had the highest Ca content, followed by those from Shanghai and Guangdong. Chongqing had the highest K content, followed by Yunan and Anhui. Yunan had the highest content of Mg, followed by Fujian and Hunan. Hubei had the highest content of P, followed by Shanghai and Hunan. Other mineral elements including Na, Mn, Fe, Cu and Zn also had significant differences (*p* < 0.05) among samples, with contents varying from 97.68 to 253.96 mg/kg, 11.69 to 325.95 mg/kg, 17.90 to 75.76 mg/kg, 1.29 to 48.42 mg/kg and 5.62 to 53.07 mg/kg, respectively. The contents of As and Se were extremely lower than the others (10- to 10,000-fold variation), with no significant differences among samples.

On the other hand, the contents of three heavy metals, Cd, Hg and Pb in CCSKs, ranged from 0.02 to 0.10 mg/kg, 0.00 to 0.04 mg/kg and 0.05 to 0.16 mg/kg, respectively (Table 4). The three heavy metals are toxic elements and have adverse effects on human health [27]. Except for Sichuan and Yunan, the contents of Cd, Hg and Pb in CCSKs met the requirements of Chinese National Standard GB2762—2017, which stipulates that the maximum contaminant levels of Cd, Hg and Pb in food are 0.5 mg/kg, 0.02 mg/kg and 0.2 mg/kg, respectively. Overall, these results indicated that CCSK consumption may significantly contribute to the daily intake of essential minerals.

### 3.4. Phytochemical Composition

**Figure 3:**
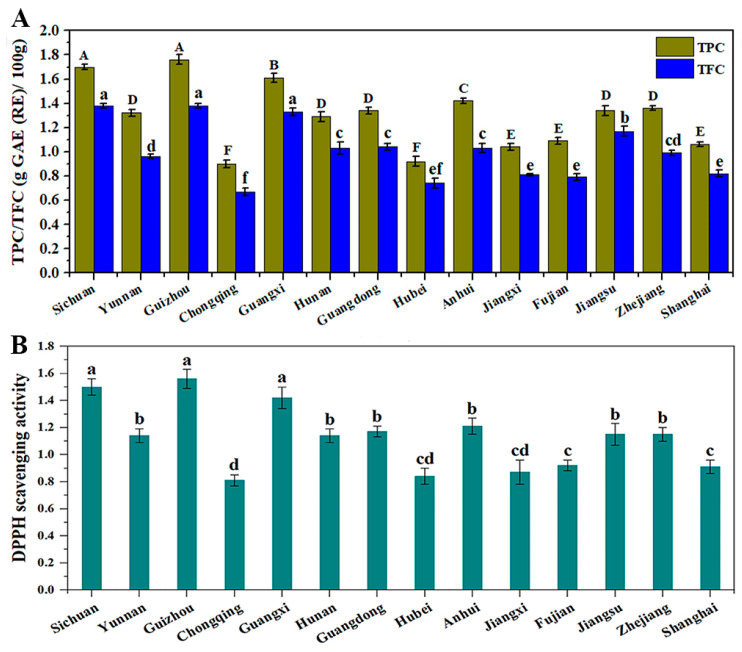
Phytochemical composition of CCSK samples collected from different geographical regions. (**A**): total phenolic content (TPC) and total flavonoid content (TFC). (**B**): DPPH scavenging activity (mmol TE/g extract) of the ethanol extract of CCSKs. CCSK: *C. camphora* seed kernel. Values with different letters ^A–E^ and ^a–f^ were significantly different (*p* < 0.05).

CCSKs exhibited antioxidant and anti-inflammatory activities mainly owing to the presence of phytochemical constituents, especially phenolics and flavonoids [2,11]. The total phenolic and flavonoid contents (TPC and TFC, respectively) in CCSKs collected from different geographical regions are shown in Figure 3A. There were significant differences (*p* < 0.05) for the TPC and TFC in CCSK samples. CCSKs collected from Guizhou had the highest TPC and TFC (1.76 g GAE/100 g and 1.38 g RE/100 g, respectively), followed by those from Sichuan (1.70 g GAE/100 g of TPC and 1.37 g RE/100 g of TFC). The lowest TPC and TFC were found in Chongqing (0.90 g GAE/100 g and 0.67 g RE/100 g, respectively). In a previous study, the TPC in CCSKs was determined to be 0.97 g GAE/100 g [3]. The HPLC showed a multimodal pattern (Figure S1), indicating that the ethanol extract of CCSKs was rich in various phytochemical components. However, CCSK samples collected from different geographical regions had similar HPLC profiles because the peak positions were basically the same.

### 3.5. Antioxidant Activity

The antioxidant activity of the phenolic extract of CCSKs was determined via DPPH radical scavenging activity (Figure 3B). The highest antioxidant activity was found in the CCSK sample collected from Guizhou (1.56 mmol TE/g extract), followed by those from Sichuan (1.50 mmol TE/g extract) and Guangxi (1.42 mmol TE/g extract). The CCSK sample collected from Chongqing had the lowest antioxidant activity with the value of 0.81 mmol TE/g extract. It can be observed that the DPPH radical scavenging activity was highly correlated with the TPC (r = 0.995) and TFC (r = 0.974) of the CCSK samples (Table S1). Therefore, the higher contents of TPC and TFC in the CCSK samples are helpful to improve their higher antioxidant activity. A similar result was also reported by Fu et al. [28], who found that the DPPH radical scavenging activity of Chinese wild raspberry (*Rubus hirsutus* Thunb.) had strong positive correlation with TPC (r = 0.847) and TFC (r = 0.852). Additionally, the DPPH radical scavenging activity of the phenolic extract of CCSKs was significantly higher than that of different cultivars of lentils (*Lens culinaris*) [29] and wheats (*Triticum* spp.) [30]. Therefore, the phenolic extract of CCSKs has great potential for development in the food, pharmaceutical and cosmetic industries.

### 3.6. Fatty Acid Composition

A total of nine fatty acids (FAs) were determined in the oil fraction of CCSKs (Table 5). Among them, capric acid (CA, C10:0), lauric acid (LA, C12:0) and oleic acid (OA, C18:1) were the major FAs in the oil, and composed approximately 97.41% to 98.63% of the total FA content: CA ranged from 57.37% to 60.18%; LA from 35.23% to 38.29%; and OA from 2.34% to 2.51%. The contents of caprylic acid (CLA, C8:0), myristic acid (MA, C14:0), palmitic acid (PA, C16:0), stearic acid (SA, C18:0), linoleic acid (LA, C18:2) and icosanoic acid (IA, C20:0) were relatively low, with no significant differences between the samples. The CA content was higher than those reported (53.27% and 54.40%) by Hu et al. [31] and Zeng et al. [18], respectively. However, the contents of LA and OA in this study were lower than those reported. Our results showed that the highest CA was in Yunan, while the lowest content was in Zhejiang. On the contrary, Zhejiang had the highest content of LA, and Yunnan had the lowest content. The contents of unsaturated fatty acids (USFAs) were low in all CCSK oils, ranging from 2.52% to 2.80% of the total FA content.

It was found that the FA of CCSK oil was mainly contributed by saturated fatty acids (SFAs, from 95.68% to 97.52%). Specially, MCFA, including CLA, CA and LA in CCSK oil, accounted for more than 94.61% of the total FA. Moreover, the MCFA content in CCSK oil was significantly higher than those of commercial coconut oil (62%) [32] and palm kernel oil (54%) [33]. The main MCFA component of coconut oil and palm kernel oil is LA, accounting for 47.58% and 47.73% of the total FA, respectively. Thus, CCSK oil could be regarded as a natural resource of medium-chain triglycerides (MCT).

Our previous studies have found that CCSK oil effectively decreased the body weight and fat deposition in healthy and obese rats compared with lard and soybean oil [9,10,22]. CCSK oil has also been widely used to prepare medium- and long-chain triacylglycerol oil (MLCT) [34,35,36]. Our recent study also proved that CCSK oil did not have acute oral toxicity in mice, with an LD50 value higher than 21.5 g/kg body weight [8]. Therefore, CCSKs may have great potential in the edible oil market.

### 3.7. Amino Acid Composition

The amino acid profiles play a key role in protein quality [37]. A total of 17 amino acids, including 8 essential amino acids (EAA) and 9 non-essential amino acids (NEAA), were determined in CCSK samples (Table 6). For EAA, leucine (Leu) was the most abundant in CCSKs, which ranged from 0.92 to 1.35 g/100 g, followed by lysine (Lys, 0.69 to 1.12 g/100 g) and phenylalanine (Phe, 0.59 to 0.93 g/100 g). The roles of Leu include repairing muscles, improving glucose and lipid homeostasis and providing energy to the body [38]. The average total EAA (TEAA) of CCSK samples was 4.41 g/100 g. The CCSKs collected from Jiangxi had the highest TEAA content (5.68 g/100 g), followed by those from Shanghai (5.26 g/100 g) and Guangdong (4.96 g/100 g), and those from Yunan had the lowest content (3.87 g/100 g). For NEAA, glutamic acid (Glu) was found to be the highest in CCSKs, ranging from 1.87 to 3.09 g/100 g, followed by arginine (Arg, 1.05 to 1.81 g/100 g) and aspartic acid (Asp, 1.18 to 2.01 g/100 g). Similar to palm, flaxseed and most grain legumes (e.g., soybean, lentil, faba bean, pea, chickpea and common bean) [39,40,41,42], Glu, Arg and Asp were present as the main amino acids in these seeds and kernels. Glu is essential for most homeostatic functions in the human body, and Arg has remarkable metabolic and regulatory versatility [43].

The total amino acids (TAA) of CCSK samples collected in different geographical regions also varied significantly (from 11.04 to 17.25 g/100 g). Shanghai had the highest TAA value, while Yunan was the lowest. In addition, the ratios of TEAA/TAA in CCSK samples varied from 28.84 g/100 g (Guangdong) to 36.20 g/100 g (Guizhou), with the average value of 31.25 g/100 g. It is one of the important scores to evaluate the nutritional value of protein ingredients. The value was lower than those of whey protein isolate and sodium caseinate, but was close to those of soybean and flaxseed protein isolates [42].

### 3.8. Protein Profile

**Figure 4:**
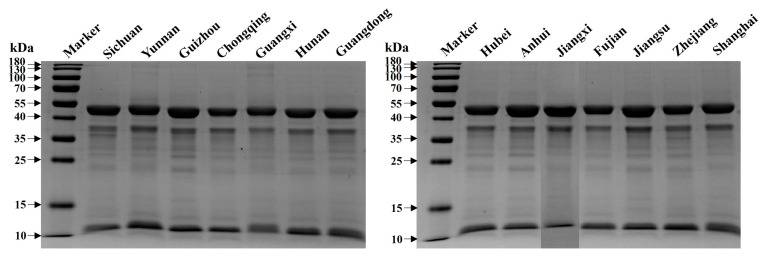
SDS-PAGE profiles of proteins isolated from CCSK samples collected from different geographical regions. CCSK: *C. camphora* seed kernel.

As shown in Figure 4, the SDS-PAGE analysis revealed that there was no significant difference in the banding pattern of proteins among CCSK samples. The band with a molecular weight (MW) between 40 and 55 kDa was the most abundant polypeptide for all samples, followed by the band with an MW between 35 and 40 kDa. Apart from these, numerous polypeptides with MWs around 10 kDa, 25 kDa and 35 kDa were observed. These results are similar with our previous study [3,44], where the protein has been shown to have excellent solubility, foaming and emulsifying properties, as well as some antioxidant properties.

### 3.9. Principal Component Analysis

**Figure 5:**
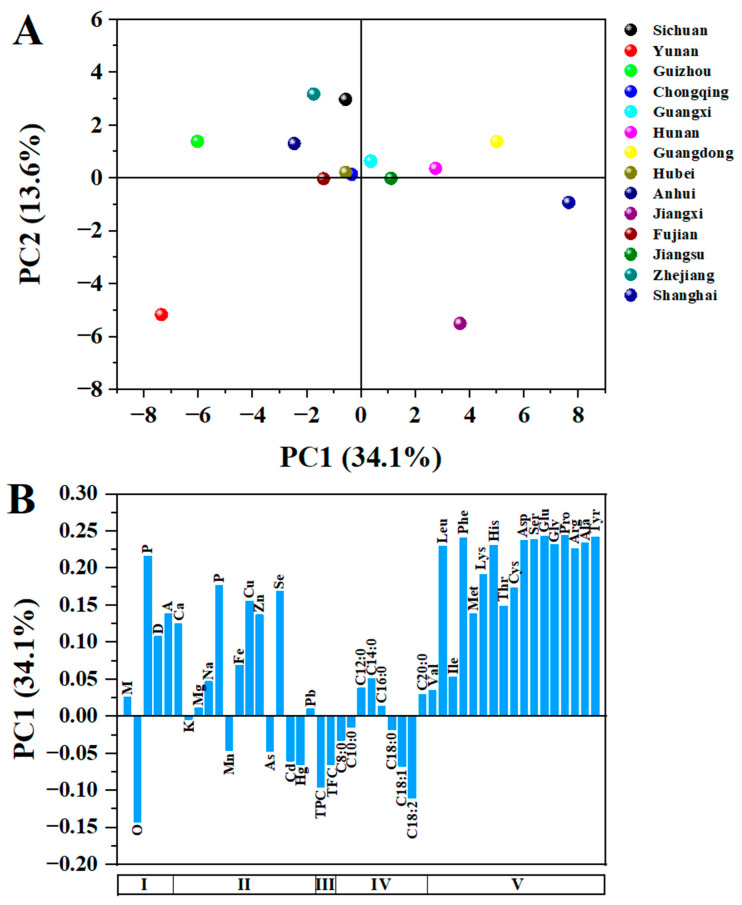
Scores plot (**A**) and loadings plot (**B**) of principal component analysis. (**B**): I—proximate composition (M: moisture, O: oil, P: protein, D: dietary fiber; A: ash); II—minerals and heavy metals; III—phytochemical constituents (TPC: total phenolic content, TFC: total flavonoid content); IV—fatty acids; V—amino acids.

The principal component analysis (PCA) can be used to visualize the differences and similarities in the chemical properties among the fourteen CCSK samples collected from different geographical regions. As shown in Figure 5A, the two extracted principal components (PC1 and PC2) explained 47.7% of the total variance. Most of the CCSK samples were in the positive part of PC2, and only CCSK samples from Yunan, Jiangxi and Anhui appeared on the negative part of PC2. Eight and six CCSK samples were located in the negative and positive parts of PC1, respectively. Therefore, PC1 (34.1%) was responsible for recognizing significant differences between CCSK samples.

To identify the variable contributions of different parts (from I to V) for the PC1 formation, the loadings plot of the PC1 was used (Figure 5B). Variables with higher contributions had larger absolute values, and variable loadings with opposite signs indicated that they were inversely correlated. CCSK samples from Guangxi, Jiangsu, Hunan, Jiangxi, Guangdong and Shanghai had positive values for PC1, which were positively correlated with protein, dietary fiber, ash, some minerals (P, Fe, Cu, Zn and Se) and amino acids. Analogously, CCSK samples from Yunan, Guizhou, Anhui, Zhejiang, Fujian, Sichuan, Hubei and Chongqing were positively correlated with oil, heavy metals, TPC, TFC and some fatty acids (C18:2 and C18:1). The values of moisture, K, Mg, Na, Pb, some fatty acids and some amino acids were close to zero, which had little correlation with CCSK samples. In summary, the PCA results also showed that geographic differences had an obvious impact on the chemical and nutritional characterization of CCSKs.

## 4. Conclusions

In this study, the physical characteristics, chemical compositions and nutritional values of fourteen CCSK samples collected from different geographical regions in China were investigated. The results showed that the weights (1000 fruits, 1000 seeds and 1000 kernels), proximate compositions, minerals, phenolics, flavonoids and amino acid contents were significantly affected by geographical factors, such as average altitude and weather conditions. Proximate composition analysis found that CCSKs had the high contents of oil (46.29–62.08%), protein (12.33–22.17%) and dietary fiber (5.66–8.03%), among which Chongqing, Shanghai and Fujian had the highest contents of oil, protein and dietary fiber, respectively. The main minerals in the CCSK samples were K (4345.05–7186.89 mg/kg), P (2735.86–5385.36 mg/kg), Ca (1412.27–3327.37 mg/kg) and Mg (2028.65–3147.32 mg/kg). The highest TPC and TFC values were found in Guizhou (1.76 g GAE/100 g and 1.38 g RE/100 g, respectively). The ratio of essential amino acids to total amino acids in the CCSK sample from Guizhou was the highest (36.20%). The antioxidant activity varied greatly with the sample location, and was highly correlated with TPC and TFC. The results suggested that CCSKs were rich in phytochemical components and had high nutritional value, and may be suitable to be used as ingredients for the food industry. However, given the heavy metal content in CCSKs, great caution should be exercised.

## Figures and Tables

**Table 1:** Sample locations of *C. camphora* in China.

No.	Geographical Regions			Coordinates
	Province	City	County/District	
1	Sichuan	Chengdu	Wuhou	104°06′ E/30°38′ N
2	Yunnan	Qujing	Xuanwei	104°08′ E/26°14′ N
3	Guizhou	Bijie	Nayong	105°23′ E/26°47′ N
4	Chongqing	Chongqing	Shapingba	106°29′ E/29°34′ N
5	Guangxi	Chongzuo	Jiangzhou	107°24′ E/22°24′ N
6	Hunan	Changsha	Tianxin	113°02′ E/28°08′ N
7	Guangdong	Shenzhen	Bao’an	113°56′ E/22°42′ N
8	Hubei	Xianning	Xian’an	114°18′ E/29°52′ N
9	Anhui	Fuyang	Yingdong	115°56′ E/32°52′ N
10	Jiangxi	Nanchang	Qingshanhu	115°58′ E/28°40′ N
11	Fujian	Xiamen	Huli	118°08′ E/24°30′ N
12	Jiangsu	Nanjing	Jianye	118°46′ E/32°02′ N
13	Zhejiang	Wenzhou	Rui’an	120°28′ E/27°50′ N
14	Shanghai	Shanghai	Pudong	121°33′ E/31°09′ N

**Table 2:** The 1000-fruit (wet, W1), -seed (wet, W2; dry, W3) and -kernel (dry, W4) weights of *C. camphora* samples collected from different geographical regions.

Sample	W1 (g)	W2 (g)	W3 (g)	W4 (g)	R1 ^a^ (%)	R2 (%)
Sichuan	650.90 ± 9.17 ^cd^	241.47 ± 4.97 ^b^	155.47 ± 4.01 ^cd^	114.04 ± 2.54 ^a^	37.10 ± 0.75 ^abc^	73.36 ± 0.54 ^bc^
Yunnan	387.80 ± 6.61 ^g^	114.57 ± 5.54 ^g^	74.35 ± 1.11 ^i^	42.97 ± 0.74 ^h^	29.53 ± 0.99 ^f^	57.80 ± 1.54 ^efgh^
Guizhou	453.77 ± 8.56 ^f^	160.07 ± 3.36 ^e^	115.77 ± 4.64 ^g^	92.25 ± 1.89 ^e^	35.28 ± 0.69 ^bcde^	79.76 ± 3.37 ^a^
Chongqing	562.33 ± 20.60 ^e^	202.67 ± 7.64 ^c^	145.23 ± 5.65 ^de^	81.15 ± 1.57 ^f^	36.04 ± 0.77 ^bcde^	55.90 ± 1.10 ^fgh^
Guangxi	761.90 ± 7.64 ^b^	262.90 ± 9.40 ^a^	189.60 ± 7.10 ^a^	99.34 ± 0.95 ^bcd^	34.51 ± 1.29 ^e^	52.46 ± 2.47 ^hi^
Hunan	339.03 ± 9.58 ^h^	122.53 ± 7.20 ^fg^	81.77 ± 1.36 ^i^	61.48 ± 0.90 ^g^	36.12 ± 1.14 ^bcde^	75.19 ± 0.92 ^abc^
Guangdong	896.87 ± 14.89 ^a^	250.17 ± 6.63 ^ab^	171.73 ± 3.06 ^b^	81.68 ± 1.18 ^f^	27.89 ± 0.35 ^f^	47.57 ± 0.56 ^i^
Hubei	477.53 ± 8.32 ^f^	134.20 ± 2.52 ^f^	101.73 ± 3.56 ^h^	62.44 ± 0.55 ^g^	28.11 ± 0.58 ^f^	61.41 ± 1.62 ^def^
Anhui	550.50 ± 23.48 ^e^	215.45 ± 4.52 ^c^	146.67 ± 5.38 ^de^	92.03 ± 2.08 ^e^	39.15 ± 0.87 ^a^	62.77 ± 0.91 ^de^
Jiangxi	536.07 ± 17.26 ^e^	183.03 ± 7.12 ^d^	131.90 ± 2.26 ^f^	100.99 ± 2.19 ^b^	34.14 ± 0.27 ^de^	76.57 ± 1.62 ^ab^
Fujian	692.07 ± 11.55 ^c^	240.20 ± 3.50 ^b^	164.13 ± 3.98 ^bc^	95.67 ± 0.98 ^cde^	34.71 ± 0.47 ^cde^	58.32 ± 1.99 ^defg^
Jiangsu	539.07 ± 16.73 ^e^	201.30 ± 2.21 ^c^	136.13 ± 4.90 ^ef^	95.00 ± 1.44 ^de^	37.36 ± 0.85 ^ab^	69.82 ± 1.51 ^c^
Zhejiang	645.43 ± 18.90 ^d^	236.33 ± 6.35 ^b^	157.37 ± 3.39 ^cd^	100.05 ± 3.28 ^bc^	36.62 ± 0.23 ^abcd^	63.60 ± 2.57 ^d^
Shanghai	477.03 ± 13.85 ^f^	171.93 ± 7.03 ^de^	123.80 ± 3.56 ^fg^	65.33 ± 2.46 ^g^	36.03 ± 0.43 ^bcde^	52.84 ± 3.55 ^ghi^

^a^ R1 (%) = (W2 × 100%)/W1; R2 (%) = (W4 × 100%)/W3. Values with different letters ^a–i^ in the same column were significantly different (*p* < 0.05).

**Table 3:** Proximate composition (g/100 g) of CCSK samples collected from different geographical regions.

Sample	Moisture	Oil	Protein	Dietary Fiber	Ash
Sichuan	2.43 ± 0.06 ^def^	53.90 ± 1.15 ^bcde^	17.86 ± 0.23 ^c^	6.65 ± 0.21 ^de^	2.32 ± 0.09 ^efg^
Yunnan	2.35 ± 0.08 ^ef^	54.83 ± 1.85 ^bcde^	12.33 ± 0.32 ^f^	6.93 ± 0.27 ^cde^	2.24 ± 0.08 ^fgh^
Guizhou	1.94 ± 0.07 ^g^	57.89 ± 1.63 ^ab^	14.38 ± 0.22 ^f^	6.92 ± 0.19 ^cde^	2.08 ± 0.04 ^h^
Chongqing	3.28 ± 0.12 ^a^	62.08 ± 2.13 ^a^	18.36 ± 0.27 ^c^	5.66 ± 0.18 ^f^	2.53 ± 0.06 ^cd^
Guangxi	2.32 ± 0.05 ^ef^	54.02 ± 1.48 ^bcde^	19.48 ± 0.21 ^b^	7.13 ± 0.34 ^bcde^	2.30 ± 0.06 ^efg^
Hunan	2.32 ± 0.04 ^ef^	46.29 ± 1.30 ^f^	16.53 ± 0.25 ^e^	6.95 ± 0.08 ^cde^	2.38 ± 0.04 ^def^
Guangdong	2.26 ± 0.08 ^f^	50.33 ± 1.64 ^ef^	20.07 ± 0.40 ^b^	7.58 ± 0.41 ^abc^	2.15 ± 0.04 ^gh^
Hubei	2.89 ± 0.07 ^b^	55.79 ± 1.40 ^bc^	17.81 ± 0.16 ^c^	6.91 ± 0.26 ^cde^	2.63 ± 0.08 ^bc^
Anhui	2.79 ± 0.11 ^bc^	61.79 ± 0.84 ^a^	16.93 ± 0.37 ^d^	6.36 ± 0.12 ^ef^	2.29 ± 0.05 ^efg^
Jiangxi	1.62 ± 0.08 ^h^	53.15 ± 1.18 ^cde^	18.35 ± 0.14 ^d^	7.84 ± 0.22 ^ab^	2.96 ± 0.08 ^a^
Fujian	2.55 ± 0.11 ^de^	55.27 ± 2.18 ^bcd^	16.31 ± 0.33 ^d^	8.03 ± 0.30 ^a^	2.45 ± 0.06 ^cde^
Jiangsu	2.87 ± 0.05 ^b^	50.93 ± 1.66 ^def^	19.83 ± 0.38 ^b^	7.31 ± 0.17 ^abcd^	2.41 ± 0.06 ^def^
Zhejiang	2.58 ± 0.06 ^cd^	56.88 ± 1.52 ^bc^	18.11 ± 0.32 ^c^	7.07 ± 0.21 ^bcde^	2.31 ± 0.03 ^efg^
Shanghai	3.00 ± 0.09 ^b^	51.04 ± 1.08 ^def^	22.17 ± 0.15 ^a^	7.80 ± 0.43 ^ab^	2.76 ± 0.09 ^b^

Values with different letters ^a–h^ in the same column were significantly different (*p* < 0.05).

**Table 4:** Mineral and heavy metal composition (mg/kg) in CCSK samples collected from different geographical regions.

Sample	Ca	K	Mg	Na	P	Mn	Fe	Cu	Zn	As	Se	Cd	Hg	Pb
Sichuan	2895.71	4347.44	2158.52	97.68	3213.95	325.95	47.93	39.76	10.85	0.01	0.50	0.05	0.03	0.16
Yunnan	2726.93	7171.88	3147.32	161.68	2735.86	11.69	17.90	4.33	7.58	0.00	0.12	0.02	0.04	0.08
Guizhou	1412.27	4798.14	2298.14	140.37	3382.76	220.01	53.92	27.53	9.92	0.02	0.33	0.07	0.00	0.08
Chongqing	1632.25	7186.89	2651.85	253.96	4116.05	30.26	75.76	27.52	9.45	0.01	0.56	0.02	0.00	0.07
Guangxi	1661.46	4345.05	2028.65	150.98	4195.96	20.87	39.49	30.23	13.03	0.01	0.40	0.02	0.01	0.13
Hunan	4573.18	6307.44	2996.73	120.77	4545.38	115.34	60.15	43.07	16.75	0.01	0.76	0.04	0.01	0.13
Guangdong	3264.21	4667.47	2457.68	237.18	4230.96	139.66	41.62	40.29	53.07	0.01	0.56	0.05	0.00	0.08
Hubei	2793.24	5252.61	2976.67	106.36	5385.36	20.72	43.39	43.31	23.27	0.01	0.53	0.01	0.01	0.16
Anhui	1975.02	7052.99	2369.42	173.43	4068.13	106.35	48.46	1.29	5.62	0.15	0.08	0.04	0.00	0.29
Jiangxi	2915.32	6534.95	2703.63	113.17	4385.75	14.97	23.93	15.40	16.19	0.00	0.11	0.02	0.01	0.07
Fujian	2666.44	5067.79	3128.19	106.98	4009.40	83.22	65.30	22.83	17.82	0.01	0.41	0.04	0.00	0.11
Jiangsu	2063.66	6350.85	2563.29	107.99	4033.31	13.86	54.56	32.75	11.48	0.01	0.45	0.04	0.01	0.14
Zhejiang	2867.80	4538.61	2198.30	105.24	4094.90	123.71	48.40	19.68	14.86	0.01	0.24	0.10	0.00	0.05
Shanghai	3327.37	6301.79	2869.57	181.24	5211.42	31.70	68.61	48.42	19.28	0.01	1.63	0.02	0.01	0.15

**Table 5:** Fatty acid composition (%) in kernel oil of CCSK samples collected from different geographical regions.

Sample	C8:0	C10:0	C12:0	C14:0	C16:0	C18:0	C18:1	C18:2	C20:0	SFA ^a^	USFA
Sichuan	0.35 ± 0.03 ^ef^	58.37 ± 0.66 ^abcd^	37.75 ± 0.63 ^ab^	0.73 ± 0.01 ^c^	0.16 ± 0.02 ^bcd^	0.07 ± 0.01 ^a^	2.51 ± 0.07 ^a^	0.17 ± 0.03 ^b^	0.00 ± 0.00 ^c^	97.43 ± 1.22 ^a^	2.68 ± 0.10 ^ab^
Yunnan	0.44 ± 0.02 ^ab^	60.18 ± 0.50 ^a^	35.23 ± 0.84 ^c^	0.83 ± 0.12 ^abc^	0.13 ± 0.01 ^d^	0.06 ± 0.01 ^a^	2.37 ± 0.07 ^a^	0.24 ± 0.03 ^ab^	0.00 ± 0.00 ^c^	96.88 ± 1.40 ^a^	2.61 ± 0.09 ^ab^
Guizhou	0.33 ± 0.01 ^f^	58.09 ± 0.36 ^bcd^	37.32 ± 0.48 ^abc^	0.78 ± 0.01 ^bc^	0.15 ± 0.01 ^cd^	0.07 ± 0.01 ^a^	2.54 ± 0.12 ^a^	0.26 ± 0.03 ^a^	0.03 ± 0.01 ^a^	96.77 ± 0.88 ^a^	2.80 ± 0.15 ^a^
Chongqing	0.42 ± 0.03 ^abcd^	58.98 ± 1.02 ^abcd^	36.81 ± 0.69 ^abc^	0.73 ± 0.02 ^c^	0.16 ± 0.01 ^abcd^	0.06 ± 0.01 ^a^	2.34 ± 0.12 ^a^	0.18 ± 0.06 ^b^	0.00 ± 0.00 ^c^	97.16 ± 1.05 ^a^	2.52 ± 0.16 ^b^
Guangxi	0.46 ± 0.01 ^a^	59.86 ± 0.79 ^ab^	35.71 ± 0.65 ^bc^	0.81 ± 0.08 ^abc^	0.16 ± 0.01 ^bcd^	0.00 ± 0.00 ^b^	2.43 ± 0.15 ^a^	0.23 ± 0.01 ^ab^	0.00 ± 0.00 ^c^	96.99 ± 0.86 ^a^	2.66 ± 0.16 ^ab^
Hunan	0.42 ± 0.01 ^abcd^	57.68 ± 0.31 ^cd^	36.51 ± 0.33 ^abc^	0.86 ± 0.03 ^abc^	0.16 ± 0.01 ^abcd^	0.05 ± 0.01 ^a^	2.46 ± 0.05 ^a^	0.19 ± 0.02 ^ab^	0.03 ± 0.01 ^a^	95.71 ± 1.08 ^a^	2.65 ± 0.06 ^ab^
Guangdong	0.40 ± 0.02 ^bcde^	58.15 ± 0.62 ^bcd^	37.62 ± 0.65 ^ab^	0.91 ± 0.04 ^ab^	0.17 ± 0.02 ^abcd^	0.05 ± 0.01 ^a^	2.39 ± 0.04 ^a^	0.20 ± 0.03 ^ab^	0.03 ± 0.01 ^a^	97.33 ± 1.32 ^a^	2.59 ± 0.07 ^ab^
Hubei	0.44 ± 0.01 ^abc^	58.84 ± 0.49 ^abcd^	36.98 ± 0.64 ^abc^	0.91 ± 0.07 ^ab^	0.19 ± 0.02 ^abc^	0.03 ± 0.00 ^ab^	2.51 ± 0.05 ^a^	0.19 ± 0.01 ^ab^	0.00 ± 0.00 ^c^	97.39 ± 1.17 ^a^	2.71 ± 0.07 ^ab^
Anhui	0.38 ± 0.03 ^cdef^	58.07 ± 1.61 ^bcd^	37.17 ± 0.77 ^abc^	0.80 ± 0.03 ^bc^	0.15 ± 0.02 ^cd^	0.07 ± 0.01 ^a^	2.52 ± 0.09 ^a^	0.22 ± 0.02 ^ab^	0.00 ± 0.00 ^c^	96.64 ± 1.35 ^a^	2.74 ± 0.10 ^ab^
Jiangxi	0.36 ± 0.01 ^def^	58.41 ± 0.16 ^abcd^	37.42 ± 0.15 ^abc^	0.92 ± 0.09 ^ab^	0.14 ± 0.02 ^d^	0.06 ± 0.01 ^a^	2.42 ± 0.06 ^a^	0.21 ± 0.01 ^ab^	0.00 ± 0.00 ^c^	97.34 ± 1.26 ^a^	2.63 ± 0.08 ^ab^
Fujian	0.44 ± 0.03 ^abc^	59.44 ± 0.23 ^abc^	36.09 ± 0.24 ^abc^	0.84 ± 0.03 ^abc^	0.20 ± 0.01 ^a^	0.08 ± 0.01 ^a^	2.46 ± 0.06 ^a^	0.21 ± 0.01 ^ab^	0.03 ± 0.01 ^a^	97.09 ± 0.96 ^a^	2.67 ± 0.07 ^ab^
Jiangsu	0.42 ± 0.01 ^abcd^	59.16 ± 0.31 ^abcd^	35.85 ± 1.48 ^bc^	0.76 ± 0.06 ^bc^	0.14 ± 0.02 ^cd^	0.06 ± 0.01 ^a^	2.40 ± 0.08 ^a^	0.20 ± 0.02 ^ab^	0.00 ± 0.00 ^c^	96.40 ± 1.03 ^a^	2.60 ± 0.11 ^ab^
Zhejiang	0.38 ± 0.01 ^def^	57.37 ± 0.73 ^d^	38.29 ± 0.69 ^a^	0.98 ± 0.08 ^a^	0.19 ± 0.01 ^ab^	0.00 ± 0.00 ^b^	2.51 ± 0.07 ^a^	0.22 ± 0.02 ^ab^	0.00 ± 0.00 ^c^	97.20 ± 0.97 ^a^	2.73 ± 0.08 ^ab^
Shanghai	0.35 ± 0.02 ^ef^	59.66 ± 0.26 ^abc^	36.47 ± 1.05 ^abc^	0.82 ± 0.06 ^abc^	0.15 ± 0.02 ^bcd^	0.06 ± 0.01 ^a^	2.43 ± 0.06 ^a^	0.22 ± 0.01 ^ab^	0.01 ± 0.00 ^b^	97.52 ± 1.14 ^a^	2.65 ± 0.06 ^ab^

^a^ SFAs: saturated fatty acids; USFAs: unsaturated fatty acids. Values with different letters ^a–f^ in the same column were significantly different (*p* < 0.05).

**Table 6:** Amino acid composition (g/100 g) in kernel protein of CCSK samples collected from different geographical regions.

Sample	Val	Leu	Ile	Phe	Met	Lys	His	Thr	TEAA ^a^	Cys	Asp	Ser	Glu	Gly	Arg	Pro	Ala	Tyr	TAA	TEAA/TAA (%)
Sichuan	0.35	1.06	0.34	0.76	0.21	0.81	0.28	0.43	4.23	0.77	1.48	0.84	2.55	0.71	1.59	0.67	0.67	0.63	14.15	29.89
Yunnan	0.47	0.92	0.47	0.59	0.11	0.69	0.23	0.40	3.87	0.51	1.05	0.61	1.87	0.50	1.18	0.46	0.51	0.47	11.04	35.05
Guizhou	0.49	0.96	0.43	0.63	0.17	0.76	0.24	0.50	4.17	0.61	1.07	0.69	1.92	0.47	1.08	0.43	0.60	0.47	11.52	36.20
Chongqing	0.32	0.99	0.29	0.71	0.20	0.85	0.27	0.38	4.00	0.83	1.53	0.76	2.52	0.71	1.57	0.66	0.67	0.60	13.87	28.84
Guangxi	0.39	1.10	0.32	0.77	0.23	0.91	0.27	0.44	4.43	0.90	1.48	0.82	2.51	0.72	1.70	0.73	0.67	0.63	14.60	30.34
Hunan	0.35	1.14	0.39	0.80	0.21	0.90	0.32	0.47	4.58	0.75	1.58	0.92	2.72	0.75	1.70	0.70	0.72	0.67	15.08	30.37
Guangdong	0.42	1.30	0.44	0.87	0.24	0.87	0.31	0.51	4.96	0.91	1.66	0.99	3.00	0.79	1.89	0.79	0.75	0.72	16.48	30.10
Hubei	0.34	1.01	0.31	0.69	0.20	0.77	0.25	0.42	3.99	0.76	1.37	0.76	2.28	0.65	1.51	0.65	0.63	0.55	13.16	30.32
Anhui	0.33	1.04	0.34	0.70	0.18	0.88	0.27	0.42	4.16	0.79	1.44	0.72	2.41	0.68	1.45	0.67	0.66	0.54	13.53	30.75
Jiangxi	0.73	1.30	0.68	0.85	0.12	1.12	0.32	0.57	5.68	1.00	1.53	0.85	2.72	0.70	1.86	0.73	0.69	0.73	16.48	34.47
Fujian	0.32	1.02	0.35	0.66	0.20	0.82	0.25	0.43	4.05	0.79	1.32	0.79	2.38	0.63	1.48	0.67	0.60	0.55	13.26	30.54
Jiangsu	0.36	1.09	0.33	0.76	0.23	0.87	0.28	0.45	4.37	0.92	1.53	0.86	2.55	0.73	1.63	0.74	0.69	0.62	14.65	29.83
Zhejiang	0.32	1.00	0.31	0.70	0.21	0.81	0.25	0.41	4.01	0.79	1.35	0.77	2.32	0.66	1.46	0.66	0.63	0.57	13.22	30.33
Shanghai	0.43	1.35	0.43	0.93	0.24	1.00	0.35	0.53	5.26	0.78	1.81	1.04	3.09	0.86	2.01	0.79	0.82	0.79	17.25	30.49

^a^ TEAA: total essential amino acids; TAA: total amino acids.

## Data Availability

Data will be made available on request.

## Supplementary Materials

The following supporting information can be downloaded at: https://www.mdpi.com/article/10.3390/foods12132630/s1, Table S1: Correlation coefficients (r) between the parameters TPC, TFC and DPPH; Figure S1: HPLC profile of the ethanol extract of CCSK samples, recorded at 280 nm.
