# Supplementary material for: Geographic Pattern of Variations in Chemical Composition and Nutritional Value of Cinnamomum camphora Seed Kernels from China
Source: Foods. 2023 Jul 7;12(13):2630. doi: 10.3390/foods12132630 (PMC10340189; doi:10.3390/foods12132630)
Supplement: Supplementary file 1 [file foods-12-02630-s001.zip › foods-2463953-supplementary.pdf]

Supplementary Material

Table S1. Correlation coefficients (r) between the parameters TPC, TFC, and DPPH.

	TPC	TFC	DPPH
TPC	1	-	-
TFC	0.970	1	-
DPPH	0.995	0.974	1

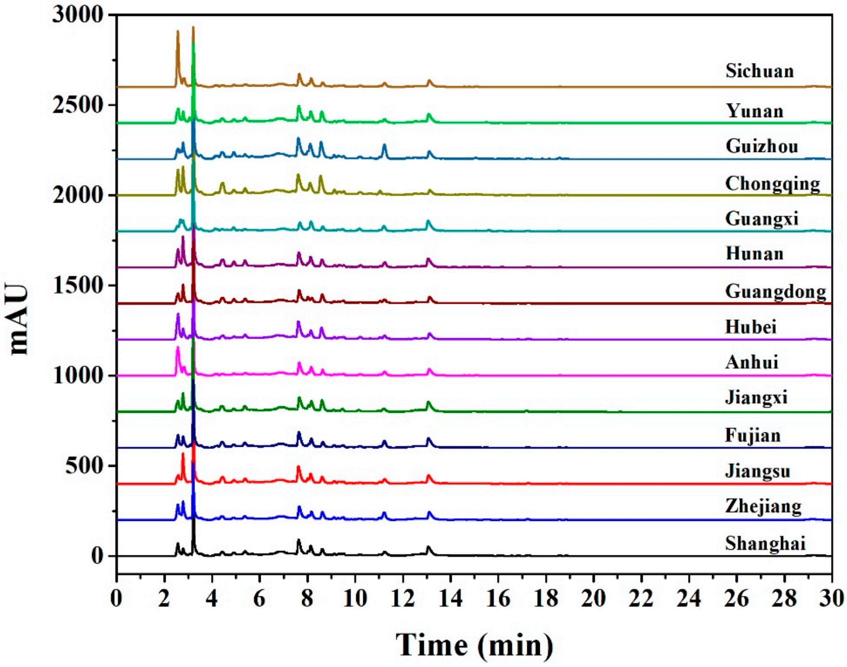

Figure S1. HPLC profile of the ethanol extract of CCSK samples, recorded at 280 nm.
